# A C-circle assay for detection of alternative lengthening of telomere activity in FFPE tissue

**DOI:** 10.1016/j.xpro.2021.100569

**Published:** 2021-05-31

**Authors:** Aurora I. Idilli, Sandra Segura-Bayona, Timothy P. Lippert, Simon J. Boulton

**Affiliations:** 1 The Francis Crick Institute, 1 Midland Road, London NW1 1AT, UK; 2 Present address: Department of Cancer Biology, Penn Center for Genome Integrity, Perelman School of Medicine, University of Pennsylvania, 421 Curie Boulevard, Philadelphia, PA 19104, USA

**Keywords:** Cancer, Molecular Biology

## Abstract

Alternative lengthening of telomeres (ALT) is a telomerase-independent, recombination-based telomere maintenance mechanism that allows cancer cells to acquire unlimited proliferative capacity. The C-circle assay (CCA) has emerged as the gold standard for quantitative measurement of ALT activity. Here, we present a modified CCA protocol to examine ALT activity in formalin-fixed paraffin-embedded specimens. We optimized several aspects of the procedure including genomic DNA isolation and hybridization steps, which allows for sensitive and robust quantitation of ALT activity in patient biopsies.

For complete details on the use and execution of this protocol, please refer to Lippert et al. (2021).

## Highlights

• FFPE-CCA is a C-circle assay to evaluate ALT activity in FFPE tissue specimens
• A step-by-step non-radioactive protocol for quantitative measurement of ALT activity
• Improved protocol to detect ALT in the largest available source of diagnostic material

## Graphical Abstract

**Figure:**
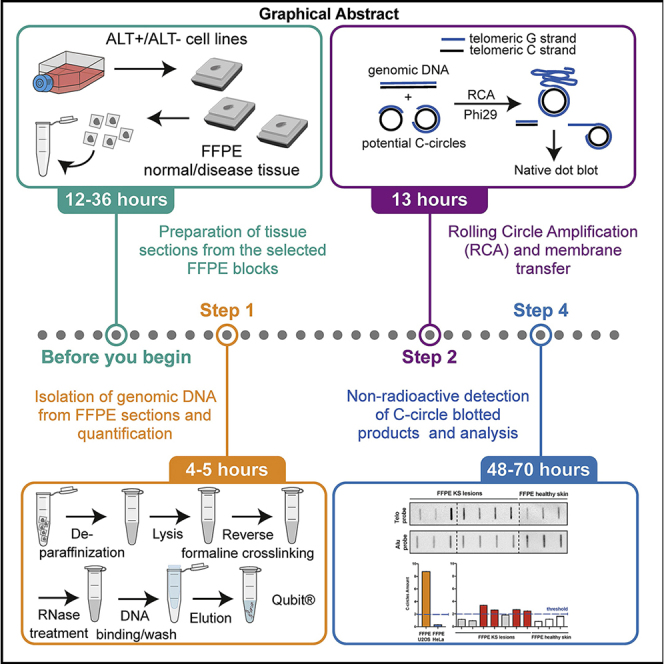

## Before you begin

The protocol described here takes several days to complete and requires the preparation of tissue sections from the selected FFPE biopsy blocks, as well as the inclusion and sectioning of FFPE control samples from established ALT+ and ALT- cell lines. Here we describe the specific steps for using biopsies from dermal Kaposi’s sarcoma (KS) lesions and a cohort of healthy skin donors. For FFPE positive and negative control samples from established ALT+ and ALT- cell lines, we used adherent U2OS (ATCC: HTB-96) and HeLa (ATCC: CCL-2) cells, respectively.

We also recommend the verification of the quality of critical reagents and the preparation of working solutions before starting the protocol:

1. Prepare working buffers. We recommend preparation in advance. All buffers can be stored for almost 6 months at 20°C–23°C or −20°C.
2. Phi29 DNA polymerase amplification efficiency is a critical step in this protocol. Make sure that there is suffcient phi29 polymerase to process all samples, and that the enzyme has been properly stored at −20°C for no longer than 24 months.
3. For maximal activity, phi29 DNA polymerase requires the presence of the reducing agent DTT in reaction buffers as well as in the reaction mixture. DTT degrades over time with temperatures higher than −20°C, multiple freeze-thaw cycles or extended storage, which can reduce amplification efficiency of phi29 DNA polymerase. The use of old reaction buffer stocks or DTT stocks that have been repeatedly frozen and thawed should be avoided.

***Note:*** Gloves and a lab coat must be worn at all times. Please use sterile disposable material and DNase-RNase free microtubes.

### Cell processing and slide preparation of FFPE cell pellets

**Timing: 2–3 days (FFPE block preparation can be done up to 1 month in advance)**

This protocol can be adapted to both FFPE cell pellets from either adherent or non-adherent cells grown in culture. Here, we describe the specific steps for using adherent U2OS and HeLa cells.

Cellular pellets should be harvested without enzymatic treatment. The protocol is suitable when starting with 7.0 ×10^6^ – 1.0 ×10^8^ cells.

***Note:*** FFPE-block preparation can be done up to 1 month in advance, but we recommend using freshly cut sections of FFPE samples as starting material for DNA extraction.

***Note:*** Care should be taken in the handling of chemicals and, if possible, they should be weighed/poured in a fume hood. Containers should have close-fitting lids.

***Note:*** Medium for tissue culture should be pre-warmed at 37°C.

4. Harvesting the cells
  a. Subculture cells 1–2 days before harvesting to achieve confluence in a 150 mm tissue-culture (TC) treated dish.
  b. Gently rinse cell monolayer with 25 mL of pre-warmed serum-free TC media (rock gently, remove and discard).
  c. Add 10 mL of warm serum-free TC media.
  d. Dislodge cells using a sterile cell scraper (“rubber policeman”).
  e. Transfer cells into a sterile, 15 mL conical centrifuge tube.
  f. Gently pellet cells from the media (centrifuge at 1000× *g*) for 5 min.

5. Fixing the cells
  ***Note:*** Use a fixation time of 16–24 h, longer fixation times can lead to severe DNA fragmentation, resulting in poor performance of the assay.
  a. Remove the supernatant from the tube leaving approximately 1 mL behind.
  b. Resuspend the cells by flicking the tube.
  c. Transfer suspended cells into a clean 1.5 mL microtube.
  d. Pellet the cells (centrifuge at 8000× *g*) for 30 s in a microcentrifuge. Discard the supernatant, leaving approximately 100–200 μL behind.
  e. Loosen pellet by gently flicking the tube and fix adding 1 mL of 10% neutral buffered formalin. Incubate pellet in fixative solution at 20°C–23°C for a minimum of 16 h, up to a maximum of 24 h.

6. Removing the fixative
  a. Pellet the cells (centrifuge at 8000× *g*) for 30 s in the microcentrifuge. Carefully discard the supernatant by pipetting if necessary, to remove all the fixative. Avoid sucking up the cells.
  b. Loosen pellet by gently flicking the tube and add 1 mL of PBS.
  c. Pellet the cells (centrifuge at 8000× *g*) for 30 s in the microcentrifuge. Carefully discard the supernatant, by pipetting if necessary, to remove all the PBS. Avoid sucking up the cells.

7. Creating the agarose pellet
  ***Note:*** Follow safety procedures and use thermo-protective gloves when removing agarose from the microwave.
  ***Note:*** The agarose gel needs to surround the pellet, but it is ideal to try to keep the cells as a pellet rather than mixing them up too much.
  a. Prepare the agarose gel (with an appropriate percentage of 1.5%–2%) by combining 0.75 g of agarose and enough ddH_2_O to reach a volume of 50 mL. Heat the solution in a microwave and create the gel (typically will take about 2 min).
  b. Leave the agarose gel to cool down for a few minutes at 20°C–23°C. The gel should have cooled substantially and reached a temperature that is safe to handle.
  c. Loosen pellet by gently flicking the tube and pipette 200–500 μL of agarose gel to pellet-containing microtube. Gently mix agarose with cells.
  d. Allow the agarose to set and then add 200 μL 70% EtOH to prevent the agarose drying out. Process the agarose pellets to embedding.

8. Sample embedding
  ***Note:*** Thoroughly dehydrate samples prior to embedding to remove residual formalin that could inhibit the subsequent proteinase K digestion during DNA isolation.
  a. Process the agarose pellets using a Tissue-Tek VIP® 6 AI and embed in CellWax Plus (CellPath GCA **030200A) ac**cording to manufacturer instructions.

### Preparation of sections from FFPE tissue specimen blocks

**Timing: 4–8 h (depending on the number of samples)**

9. Carefully check each FFPE block for the quality of the tissue samples. We recommend selecting blocks whose section has a surface area of at least 2 mm^2^ and do not exhibit tissue damage upon histological examination. This step is usually performed by a pathologist who can verify and ascertain sample adequacy after hematoxylin/eosin staining. Considering the number of tumor samples chosen, select a number of healthy samples that corresponds to at least one-third of the tumor samples.
10. Proceed with sectioning of the FFPE blocks. Sections should be freshly cut and prepared no more than 10 days before the DNA extraction (see section “Isolation of genomic DNA from FFPE sections”). FFPE sections can be prepared in the standard manner from tissue blocks. We recommend removing excess paraffin from the sample block using a scalpel. If the block surface has been exposed to air, discard the first 2–3 sections.
11. Collect 3–8 sections per sample depending on sample surface area, each section with a thickness of 5 μm in a 1.5 mL microtube. If possible, prepare at least 3 microtubes/normal and tumor sample (to perform the assay in triplicate) and 6 microtubes/control cells sample. Store at +4°C (up to 10 days) until proceeding with DNA extraction.

## Key resources table

**Table:** 

REAGENT or RESOURCE	SOURCE	IDENTIFIER
**Antibodies**		

Anti-Digoxigenin-AP, Fab fragments	Roche	Cat#11093274910 RRID:AB_2734716

**Chemicals, reptides, and recombinant proteins**		

Formalin solution, neutral buffered, 10%	Sigma-Aldrich	Cat#HT501128
Agarose	Sigma-Aldrich	Cat#A9539
Ethanol absolute 99.8+%	Fisher Scientific	Cat#E/0650DF/17
Xylene 99%	Fisher Scientific	Cat#95692
phi29 DNA polymerase (10 U/μL)	Thermo Fisher	Cat#EP0092
Sarkosyl 20%	Sigma-Aldrich	Cat#L7414
SDS 20%	Bio-Rad	Cat#1610418
Tween-20	Sigma-Aldrich	Cat#P1379
dNTP set (100 mM)	Invitrogen	Cat#10297118
DDT 1M	Thermo Fisher	Cat#P2325
Maleic acid	Sigma-Aldrich	Cat#M0375
Bovine serum albumin (BSA) 20 mg/mL	Thermo Fisher	Cat#B14
Blocking reagent	Roche	Cat#11096176001

**Critical commercial assays**		

QIAamp® DNA FFPE Tissue Kit	QIAGEN	Cat#56404
Qubit® dsDNA HS Assay Kit	Life Technologies	Cat#Q32851
DIG Oligonucleotide 3’-End Labeling Kit, 2nd generation	Roche	Cat#03353575910

**Experimental models/cell lines**		

U2OS cells	ATCC	HTB-96
HeLa cells	ATCC	CCL-2
FFPE normal human skin	The Francis Crick Institute	HTA #12650
FFPE KS samples	Imperial College Healthcare Tissue Bank	HTA #12275

**Oligonucleotides**		

Telomere probe oligo 5’ –CTAACCCTAACCCTAACC-3’	Sigma-Aldrich	N/A
Alu probe 5’-GTAATCCCAGCACTTTGG-3’	Sigma-Aldrich	N/A

**Software and algorithms**		

Image Lab Software	Bio-Rad	RRID:SCR_014210

**Other**		

Hybond-N+ (11.9 × 7.8 cm) membrane	Amersham	Cat#RPN119B
Qubit 4 Fluorometer	Life Technologies	Cat#Q33238
Qubit® assay tubes (500 tubes)	Life Technologies	Cat#Q32856
Thermoshaker	N/A	N/A
Vortex	N/A	N/A
UV cross-linker	N/A	N/A
Hybridization oven (type: rotating, temperature range: ≥ 70°C)	N/A	N/A
Hybridization tube (35 × >150mm)	N/A	N/A
Microcentrifuge for PCR tubes	N/A	N/A
ChemiDoc MP Imaging System	Bio-Rad	Cat#12003154
Slot Blot Blotting Manifold	Hoefer	Cat#PR648
T100 Thermal Cycler	Bio-Rad	Cat#1861096

## Materials and equipment

***Alternatives:*** This protocol uses a Qiagen QIAamp® DNA FFPE Tissue Kit to isolate DNA from FFPE sections. As an alternative to Qiagen QIAamp® DNA FFPE Tissue Kit, other commercially available kits for FFPE DNA extraction based on DNA binding to a silica membrane may be used. We discourage using a combination of a de-paraffinization step and phenol-chloroform DNA extraction because during the precipitation step, C-circles appear more challenging to precipitate than genomic DNA. If the amount of starting material is limited, phenol-chloroform extraction results in poor C-circles yield and relative C-circle levels cannot be reliably quantitated. For samples with low amounts of starting material, the well-established Quick C-circle preparation method (Henson et al., 2017) may not be suitable. Quick C-circle preparation method (Henson et al., 2017) is a DNA isolation method for cell pellets and frozen tumor specimens. This method does not involve any step where DNA can be lost and provides higher C-circles yields than other DNA extraction methods. However, it is not indicated for over-diluted samples, where the presence of detergent used in the lysis buffer can inhibit the CCA.

***Note:*** For a reproducible comparison among various specimens, we recommend being consistent with DNA isolation method and the amount of starting material.

***Alternatives:*** This protocol uses Qubit dsDNA HS Assay Kit to quantify DNA isolated from FFPE sections. As an alternative, we recommend using high sensitivity fluorometric assays detecting DNA in a range of 10 pg/μL to 100**–**200 ng/μL. As an indication of the amount of genomic DNA typically obtained from patient samples, please refer to Figure 2.

***Alternatives:*** This protocol uses a non-radioactive chemiluminescent assay to detect C-circles in tumour and cell line samples. The blotted DNA products resulting from the rolling circle amplification (RCA) are hybridized to a digoxigenin (DIG)-labeled probe that is specific for telomeric repeats, and then incubated with a DIG-specific antibody covalently coupled to alkaline phosphatase. As an alternative to our method, equivalent commercial kits can be used i.e., TeloTAGGG telomere length kit (Roche). DIG alternatives to radioactive probes have been developed and used successfully in several assays and reduced the generation of radioactive waste and associated health risks. However, detection by radioactive probe labelling is considered more sensitive and can be used to improve extratelomeric DNA circles detection efficiency. Our DNA isolation protocol is compatible with PCR assays, and telomeric qPCR can also be used to detect C-circles products by determining the change in total telomeric signal (Lau et al., 2013).

### Experimental model and subject details

The normal human skin paraffin embedded samples used as part of this research were fully anonymized and collected before the Human Tissue Act 2004 was implemented in September 2006. These are stored under the Francis Crick Institute’s HTA license (#12650) (originally donated by NHS Whittington Hospital to the Experimental Histopathology Science Technology platform at the Francis Crick Institute). They are classed as existing holdings, therefore were able to be used for research without the need for consent. Archived FFPE-embedded tissue sections from KS patients are continuously collected, anonymized and deposited in the Imperial College Healthcare Tissue Bank (ICHTB). ICHTB is approved by the National Research Ethics Service (NRES) to release human material for research. The samples for this project are issued from a sub-collection that covers this project and has been approved by ICHTB (HTA #12275).

**Table:** Rolling Circle Master Mix (RCMM)

Reagent	Final concentration	Amount
DTT (1 M)	8.65mM	8 μL
10× phi29 Buffer	2.16×	200 μL
BSA (0.2 mg/mL)	8.65 μg/mL	40 μL
Tween-20 (10%)	0.216%	20 μL
dATC (100 mM)	2.16 mM	20 μL
dCTP (100 mM)	2.16 mM	20 μL
dGTP (100 mM)	2.16 mM	20 μL
dTTP (100 mM)	2.16 mM	20 μL
ddH_2_O DNase/RNase-free	n/a	577 μL
**Total**	**n/a**	**925 μL**

Store at −20°C up to 6 months. Avoid multiple freeze/thaws or extended storage.

Right before use, add phi29 DNA polymerase (see details in section: Rolling Circle amplification (RCA)).

**Table:** 20× SSC (pH 7.0)

Reagent	Final concentration	Amount
NaCl	3 M	175.3 g
Sodium Citrate	0.3 M	88.2 g
Autoclaved ddH_2_O	n/a	Up to 1 L
**Total**	**n/a**	**1 L**

Adjust pH to 7.0. Sterilize by autoclaving. Store at 20°C–23°C up to 1 year.

**Table:** 1 M Tris-HCl (pH 9.5) and (pH 7.5)

Reagent	Final Concentration	Amount
TRIZMA base	1 M	121,14 g
Autoclaved ddH_2_O	n/a	Up to 1 L
**Total**	**n/a**	**1 L**

Adjust pH to 9.5 and 7.5. Be sure the pH is stable. Store at at 20°C–23°C up to 6 months.

**Table:** 5× maleic acid buffer (pH 7.5)

Reagent	Final concentration	Amount
Maleic acid	0.5 M	58.05 g
NaCl	0.75 M	43.83 g
Autoclaved ddH_2_O	n/a	Up to 1 L
**Total**	**n/a**	**1 L**

Adjust pH to 7.5 adding solid NaOH. Store at 20°C–23°C up to 1 year.

**Table:** Wash buffer 1

Reagent	Final concentration	Amount
20× SSC	2×	100 mL
20% SDS	0.1%	5 mL
Autoclaved ddH_2_O	n/a	895 mL
**Total**	**n/a**	**1 L**

Store at 20°C–23°C up to 1 year.

**Table:** Wash buffer 2

Reagent	Final concentration	Amount
5× Maleic acid buffer (pH 7.5)	1×	200 mL
Tween-20	0.3%	3 mL
Autoclaved ddH_2_O	n/a	797 mL
**Total**	**n/a**	**1 L**

Store at 20°C–23°C up to 1 year.

**Table:** AP buffer

Reagent	Final concentration	Amount
1 M Tris-HCl (pH 9.5)	0.1 M	50 mL
5 M NaCl	0.1 M	10 mL
Autoclaved ddH_2_O	n/a	440 mL
**Total**	**n/a**	**500 mL**

Store at 20°C–23°C up to 1 year.

**Table:** Prehybridization buffer

Reagent	Final concentration	Amount
20× SSC	5× SSC	12.5 mL
20% Sarkosyl	0.1%	0.25 mL
20% SDS	0.04%	100 μL
Autoclaved ddH_2_O	n/a	37.25 mL
**Total**	**n/a**	**50 mL**

Prepare fresh.

**Table:** Blocking buffer

Reagent	Final concentration	Amount
Blocking reagent	1%	1 g
5× Maleic acid buffer (pH 7.5)	1×	20 mL
Autoclaved ddH_2_O	n/a	Up to 100 mL
**Total**	**n/a**	**100 mL**

Heat it to 70°C to dissolve, then cool to 20°C–23°C before use. Prepare fresh.

**Table:** Stripping neutralization buffer

Reagent	Final concentration	Amount
Tris-HCl 1 M (pH 7.5)	50 mM	25 mL
20× SSC	0.1×	2.5 mL
20% SDS	0.1%	2.5 mL
Autoclaved ddH_2_O	n/a	Up to 500 mL
**Total**	**n/a**	**500 mL**

Store at 20°C–23°C up to 1 year.

**Table:** Other solutions (prepare with autoclaved ddH_2_O)

Name	Concentration	Volume	Store (1 year)
NaCl	5 M	500 mL	20°C**–**23°C
EDTA (pH 8)	0.2 M	5 mL	20°C**–**23°C in aliquots of 500μL
NaOH	50 mM	500 mL	20°C–23°C

## Step-by-step method details

**Figure 1:**
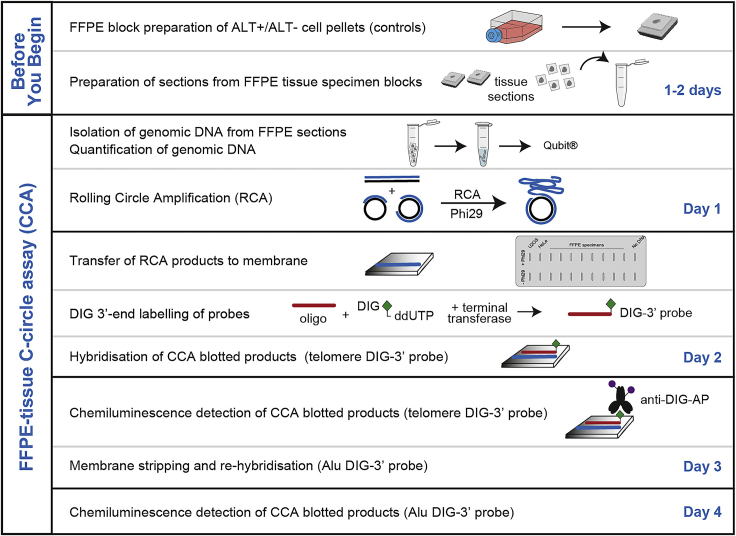
A schematic of the protocol step by step

A schematic of this protocol is shown in Figure 1.

### Isolation of genomic DNA from FFPE sections

**Timing: 3–4 h**

A protocol modification of Qiagen QIAamp® DNA FFPE Tissue Kit is used to isolate genomic DNA from FFPE sections (https://www.qiagen.com/us/resources/resourcedetail?id=67ecad65-5f6c-4559-ae24-b614180bfc4b&lang=en). This method provides a reproducible and consistent genomic DNA yield.

**Figure 2:**
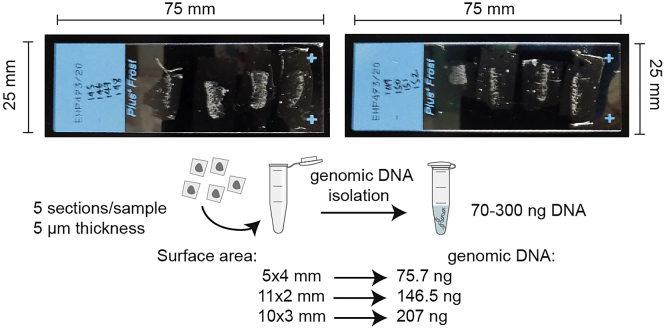
Isolation of genomic DNA from FFPE sections Example of FFPE sections used in the DNA extraction, and representative correlations between FFPE slice surface area and DNA yield obtained.

***Note:*** As starting material, we recommend using freshly cut FFPE sections with a minimum of paraffin around the samples. We recommend starting with 3-8 sections per sample, each with a thickness of 5 μm. As a maximum limit for one preparation do not exceed 8 sections with a thickness of 10 μm each and a surface area of 250 mm^2^ (Figure 2).

***Note:*** To preserve the C-circle telomeric DNA after genomic isolation, the samples should be stored at −80°C or in a −20°C freezer without a defrost cycle. Avoid multiple freeze/thaws or extended storage. During the processing, keep the samples on ice. Always use sterile disposable material and DNase-RNase free microtubes.

1. De-paraffinization of FFPE sections (all steps are performed at 20°C–25°C).
  a. Pre-heat two thermomixer devices to 37°C and 56°C.
  b. Reconstitute Buffer AW1 by adding 25 mL of ethanol (96%–100%) to the bottle containing 19 mL Buffer AW1 concentrate. Reconstitute Buffer AW2 by adding 30 mL of ethanol (96%–100%) to the bottle containing 13 mL Buffer AW2 concentrate. Mix reconstituted Buffer AW1 and AW2 by shaking. Equilibrate all the buffers at 20°C–23°C (∼15 min) and check for the presence of precipitate.
    **CRITICAL:** Do not exceed the time samples are left in xylene as required by the protocol. If needed, perform sequential DNA isolation, and process a few samples at a time.
  c. Add 1 mL of xylene to the 1.5 mL microtube containing the 3–8 FFPE sections (see section: before you begin). Close the lid and vortex vigorously for 30 s.
  d. Centrifuge at full speed (20,000× *g*; 14,000 rpm) for 2.50 min at 20°C–23°C .
  e. Carefully remove the supernatant by pipetting without removing any of the pellets.
  f. Add 1 mL of ethanol (96%–100%) to the pellet, and mix by vortexing for 30 s.
    ***Note:*** This step is essential to remove residual xylene from the sample.
  g. Centrifuge at full speed (20,000× *g*; 14,000 rpm) for 3 min at 20°C–23°C.
  h. Carefully remove the supernatant and any residual ethanol by pipetting, without removing any of the pellets.
  i. To remove the residual ethanol, open the tube and incubate at 20°C–23°C or up to 37°C until residual ethanol has evaporated (∼10 min). Do not overdry the samples.
  j. When ethanol has evaporated, remove the samples and pre-heat the thermomixer devices to 90°C.

2. Lysis under denaturing conditions with proteinase K.
  a. Resuspend the pellet in 180 μL Buffer ATL. Add 20 μL proteinase K, and mix by vortexing.
  b. Incubate at 56°C using a mixing frequency of 1400 rpm for 1 h until the sample has been completely lysed.
3. Formalin crosslink reversal.
  a. Incubate at 90°C for 1 h.

***Note:*** The incubation step at elevated temperature after proteinase K digestion allows to partially remove formalin crosslinking of the released DNA and improve DNA yields. Do not exceed the recommended temperature and incubation time. The degree of DNA fragmentation depends on the type and age of the sample and the conditions used for fixation. As reported before, we recommend to use a fixation time of 16–24 h, and discard sections that are exposed to air.

4. RNase treatment.
  a. Spin-down the 1.5 mL microtube for few s.
  b. Allow the sample to cool to 20°C–23°C (∼1–2 min) and add 2 μL of RNase A (100 mg/mL) and incubate for 2 min at 20°C–23°C.

5. Binding of DNA to membrane.
  a. Premix in a 15 mL conical tube N ∗ 200 μL of Buffer AL and N ∗ 200 μL of ethanol (96%–100%), being N the number of samples.
  b. Add 400 μL of Buffer AL+ethanol (96%–100%) mixture to the sample, and mix thoroughly by vortexing. A white precipitate may form on the addition of mixture Buffer AL and ethanol. The precipitate does not interefere with DNA extraction.
  c. Spin-down the 1.5 mL microtube for few seconds.
  d. Carefully transfer the entire lysate to the QIAamp MinElute column (in a 2 mL collection tube) without wetting the rim.
  e. Centrifuge at 6000× *g* (8000 rpm) for 1 min.
    ***Note:*** Take care when removing the QIAamp MinElute column and collection tube from the centrifuge, to avoid contact between QIAamp MinElute column and flow-through.
  f. Place the QIAamp MinElute column in a clean 2 mL collection tube, and discard the collection tube containing the flow-through. Check if the lysate has completely passed through the membrane after centrifugation, otherwise, centrifuge again until the QIAamp MinElute column is empty.
  g. Add 500 μL of Buffer AW1 without wetting the rim.
  h. Centrifuge at 6000× *g* (8000 rpm) for 1 min.
  i. Carefully, place the QIAamp MinElute column in a clean 2 mL collection tube, and discard the collection tube containing the flow-through.
  j. Add 500 μL Buffer AW2 without wetting the rim.
  k. Centrifuge at 6000× *g* (8000 rpm) for 1 min.
  l. Carefully, place the QIAamp MinElute column in a clean 2 mL collection tube, and discard the collection tube containing the flow-through.
  m. Centrifuge at full speed (20,000× *g*; 14,000 rpm) for 3 min to completely dry the membrane and remove residual ethanol.

6. DNA elution.
  a. Place the QIAamp MinElute column in a clean 1.5 mL microtube.
  b. Add 23 μL of 10 mM Tris pH 7.6 (equilibrated at 20°C–23°C) to the center of the membrane to ensure complete elution of bound DNA.
    ***Note:*** Extracted DNA should be stored in a buffer, such as 10 mM Tris pH 7.6, to avoid acid hydrolysis. Purified water has a low buffering capacity and can significantly decrease the C-Circle template. The volume of elution buffer is according to the amount of starting material and the requirements of the downstream application.
  c. Incubate at 20°C–23°C for 5 min.
  d. Centrifuge at full speed (20,000× *g*; 14,000 rpm) for 1 min.
  e. To increae DNA yield, repeat step ‘b-d’ using the eluate from step ‘d’. Reuse the collection tube from step ‘a’.
  f. Transfer the samples to ice before DNA quantification.

### Quantification of genomic DNA

**Timing: 1 h (for 20 samples)**

To quantify DNA isolated from FFPE sections we use Qubit dsDNA HS Assay Kit (https://www.thermofisher.Acom/document-connect/document-connect.html?url=https%3A%2F%2Fassets.thermofisher.com%2FTFS-Assets%2FLSG%2Fmanuals%2FQubit_dsDNA_HS_Assay_UG.pdf&title=VXNlciBHdWlkZTogUXViaXQgZHNETkEgSFMgQXNzYXkgS2l0cw==).

The CCA signal is usually reported relative to the corresponding amount of genomic DNA. This method provides an accurate and reproducible DNA sample concentration from 10 pg/μL to 100 ng/μL. The assay is performed at 20°C–23°C, and the signal is stable for 3 h.

**CRITICAL:** DNA quantification should be performed at the same time for all the samples, this includes: Tumour and Normal FFPE tissue samples, ALT+ and ALT- control FFPE cells samples. Pipetting accuracy and significant temperature fluctuations within the laboratory can impair DNA quantification outcome. For this reason, we recommend performing a new calibration each time the instrument is used.

***Note:*** Optimal performance with Qubit dsDNA HS Assay can be obtained when all the solutions are equilibrated at 22–28˚C, as temperature fluctuations can influence the accuracy of the assay. We suggest not to hold the assay tubes in your hand before reading.

7. Prepare Qubit working solution and assay tubes.
  a. Set up 1× 0.5 mL Qubit assay tubes per sample and 2× 0.5 mL Qubit assay tubes for standards.
    ***Note:*** Label only the lid of the tubes and not the side of the tube, as this could interfere with the sample reading.
  b. Prepare the Qubit working solution by diluting the Qubit dsDNA HS Reagent 1:200 in Qubit dsDNA HS Buffer in a plastic conical tube. To calculate the total volume of working solution, consider (N + 1) ∗ 200 μL, being N the number of samples, and 400 μL corresponding to standards.
    ***Note:*** Do not mix the working solution in a glass container. The final volume in each tube must be 200 μL. Each standard tube requires 190 μL of Qubit working solution plus 10 μL of each Qubit standard, and each sample tube requires from 180–199 μL of Qubit working solution plus 1–20 μL of sample.
  c. Add 190 μL of Qubit® working solution to each of the tubes used for standards.
  d. Add 10 μL of each Qubit standard to the appropriate tube (Standard # 1: 0 ng/μL; Standard # 2: 10 ng/μL) mix by vortexing 2–3 s without creating bubbles.
    ***Note:*** Carefully pipet exactly 10 μL of each Qubit standard.
  e. Add 197 μL of Qubit working solution to individual assay tubes.
  f. Add 3 μL of each sample to the assay tubes containing the volume of Qubit working solution, then mix by vortexing 2–3 s.
  g. Incubate the tubes at 20°C–23°C for 2 min.

8. Proceed to Qubit Fluorometers.
  a. On the Home screen press “DNA”, select “dsDNA High Sensitivity”, select “Read standards”, select “Reading new standards”, press to proceed.
  b. Insert the tube containing Standard #1 into the sample chamber, close the lid, and then press “Read standard”. When the reading is complete, remove Standard #1.
  c. Insert the tube containing Standard #2 into the sample chamber, close the lid, and then press “Read standard”. When the reading is complete, remove Standard #2.
  d. The instrument displays the results on the Read standard screen.
  e. Proceed with samples reading, press “Run samples”.
  f. On the assay screen, select the sample volume: 3 μL.
  g. Select the units for the output sample concentration (ng/μL).
  h. Insert a sample tube into the sample chamber, close the lid, and then press “Read tube”. When the reading is complete, remove the sample tube.
  i. The instrument displays the results; the top value is the concentration of the original sample (ng/μL). The bottom value is the dilution concentration. Troubleshooting 1
  j. Repeat step ‘h’ until all samples have been read.

**Pause point:** DNA could be stored at −80°C or in a −20°C freezer without a defrost cycle.

### Rolling circle amplification

**Timing: 12 h**

The rolling circle amplification (RCA) step is an isothermal enzymatic process where the processive phi29 DNA polymerase uses partially single-stranded telomeric (CCCTAA)n DNA circles (C-circles) as a template and amplifies them to form a long single stranded DNA concatemers containing hundreds of tandem repeats that are complementary to the circular template. RCA followed by detection by telomere specific probes has been established as a linear quantitation of ALT activity (Henson et al 2009). The protocol described here has been adapted from (Henson et al., 2017).

***Note:*** Starting material for RCA reactions can be as low as 20 ng, and we recommend no higher than 120 ng. To obtain reliable results, the same amount of genomic DNA/sample should be used. If you have no information about the nature of your starting material, start with 30 – 40 ng. If required, add a DNA pre-dilution step with 10 mM Tris pH 7.6 to obtain the desired DNA amount.

**Figure 3:**
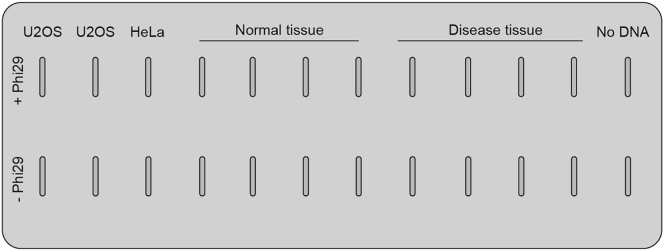
Representative layout of a slot-blot Representative layout showing an example of how to load the samples on the slot blot. An internal reference of known ALT positive and ALT negative controls is included, as well as specific background controls, where RCA reaction without phi29 polymerase is loaded in parallel to all the samples, and a single global background control, where DNA is omitted from RCA reaction.

***Note:*** RCA preparation should be done on ice.

**CRITICAL:** Since tumour and normal tissue specimens can contain significant background signal, we recommend to include the following controls in each set of reactions (Figure 3). **Specific****background controls****:** run an RCA reaction in parallel to all the samples without phi29 polymerase. When insufficient tumor sample is available to allow two reactions/sample, this control can be omitted and the evaluation of C-circles level must be corrected for CCA loading control (refer to the section: Quantification and Statistical Analysis). **Global background control****:** run a single RCA reaction where DNA is omitted. **ALT positive and ALT negative controls****:** positive (U2OS) and negative (HeLa) controls should be included in each set of reactions (i.e., 30 ng).

9. Prepare RCA reaction.
  a. Thaw Rolling Circle Master Mix (RCMM) and genomic DNA samples on ice.
  b. Dilute desired concentration of genomic DNA (i.e., 30 ng) in a total volume of 10 μL with 10 mM Tris pH 7.6. Prepare two 0.2 mL PCR tubes per each sample.
  c. Where N is number of samples:
    i. prepare RCMM without phi29 polymerase: aliquot N ∗ 10 μL ∗ 1.2 RCMM
    ii. Prepare RCMM with phi29 polymerase: aliquot N ∗ 9.25 μL ∗ 1.2 RCMM, add N ∗ 0.75 μL ∗ 1.2 of phi29 polymerase.
  d. Pipet 10 μL of RCMM (without phi29) to the first tube containing 10 μL genomic DNA sample.
  e. Pipet 10 μL of RCMM (with phi29) to the second tube containing 10 μL genomic DNA sample.
  f. Mix the resulting 20 μL RCA reactions by vortexing 5 s at 1800 rpm twice.
  g. Microcentrifuge tubes for 10 s.
  h. Incubate RCA reactions in a thermocycler using the following incubation settings:
    i. 30°C for 8 h
    ii. 70°C for 20 min
    iii. 10°C infinite hold

**Pause point:** RCA reactions can be stored at −20°C for later use.

### Transfer of RCA products to the membrane

**Timing: 1 h**

This step transfers RCA products to a membrane using a dot or slot-blot apparatus.

**CRITICAL:** Samples that will be directly compared (i.e., samples with and without phi29 polymerase, positive and negative controls) should be blotted in the same membrane (Figure 3). When we want to compare a cohort of samples larger than the ones a slot-blot membrane can fit, positive (U2OS) and negative (HeLa) controls should be included in each membrane so the signal of each specimen can be normalized relative to that of U2OS.

***Note:*** Manage the membrane with clean tweezers, do not touch with gloved hands.

***Note:*** Samples should be transferred for a native slot-blot, so RCA products can be detected. No denaturation step is needed, do not boil these samples before transferring to the membrane.

10. Transfer RCA to Nylon positively charged membrane.
  a. Prepare a Nylon positively charged membrane and two sheets of Whatman 3 MM chromatography paper, cut so it fits the slot-blotting apparatus.
  b. Soak membrane in 2× SSC buffer for 5 min.
  c. Wet Whatmann paper in 2× SSC buffer.
  d. If RCA reactions were stored frozen, thaw on ice.
  e. Add 150 μL 2× SSC to 20 μL RCA reactions.
  f. Mix the resulting 170 μL RCA reactions by vortexing 5 s.
  g. Microcentrifuge tubes for 10 s.
  h. Assemble slot-blot with two Whatman papers and membrane on top.
    ***Note:*** Ensure there are no air bubbles (can use a roller to remove bubbles). Do not allow the membrane to dry.
  i. Screw slot-blot apparatus tight and apply vacuum.
  j. Wash membrane with 200 μL 2× SSC buffer per well and let run until wells empty.
  k. Apply samples and let run until wells empty.
  l. Wash membrane with 200 μL 2× SSC buffer per well and let run until wells empty.
  m. Stop applying vacuum.
  n. Disassemble slot-blot apparatus. Place membrane, with DNA side facing upwards, onto a dry clean sheet of Whatman paper. Dry membrane for 5 min.
  o. Place the membrane in a 254 nm UV cross-linker. Cross-link DNA onto the membrane at a setting of 120 mJ/cm^2^. Flip the membrane (DNA side facing down) and repeat.
    **Pause point:** Membrane may be stored for later use. Protect the membrane in between 2 sheets of dry Whatman paper and store at 20°C–23°C.

### Non-radioactive detection of C-circle blotted products

**Timing: 48–72 h**

The CCA products are detected by native slot-blot with DIG-labeled telomere probes and corrected for CCA loading control with a DIG-labeled probe for repetitive Alu elements (Batzer and Deininger 2002). After hybridization, C-circles are detected with an anti-DIG-AP antibody conjugated to alkaline phosphatase which catalyzes the chemiluminescent reaction. Chemiluminescent alkaline phosphatase substrate (CDP-STAR) is used to detect the DIG signal in the slot-blot. The protocol described here has been adapted from (Kimura et al., 2010).

***Note:*** The volume of solutions reported in the protocol are considered for a membrane sheet of approximately 11.9 × 7.8 cm. Scale the volumes accordingly to process membranes of different sizes.

To label the telomere and Alu oligonucleotides we use the DIG oligonucleotide 3’-end labeling kit, 2nd generation (Roche) (https://www.sigmaaldrich.com/content/dam/sigma-aldrich/docs/Roche/Bulletin/1/03353575910bul.pdf). This kit uses terminal transferase to enzymatically label oligonucleotides at their 3’ ends by incorporation of a single DIG-labeled dideoxyuridine-triphosphate (DIG-ddUTP).

***Note:*** After the labelling procedure, we recommend determining the labelling efficiency of the probe. This step is essential to calculate the correct amount of probe needed for the hybridization step (12.d).

**CRITICAL:** The kit (vial 1) contains potassium cacodylate that is toxic by inhalation and if swallowed. Use gloves when handling and discard according to regulation for toxic waste.

**Figure 4:**
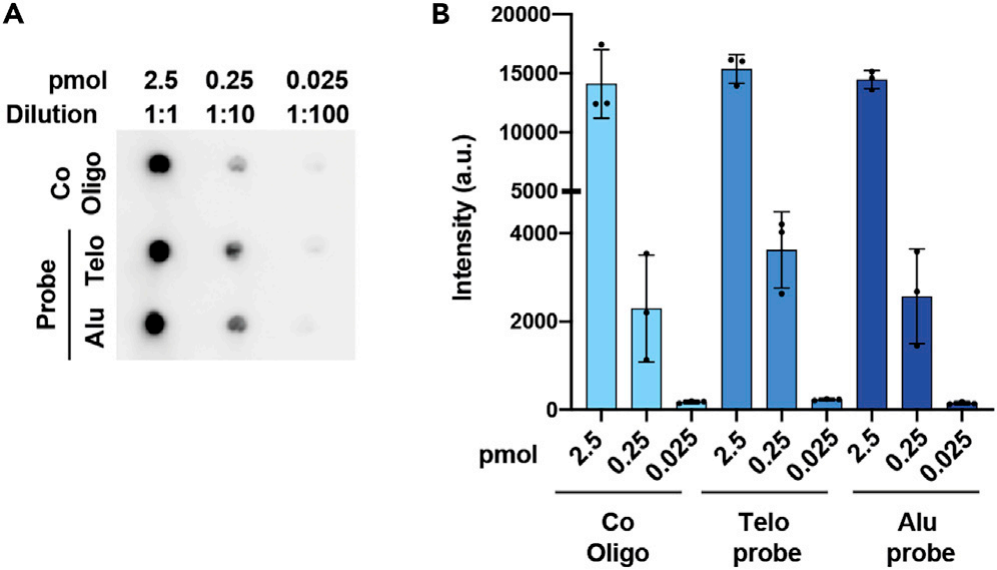
Determination of probe labeling efficiency (A and B) (A) The 3’ DIG-labeled telomere (Telo) and Alu probes were synthesized from 100 pmol of template. Serial diluitions of each probe, were spotted on a Hybond N+ positively charged nylon membrane, besides the corresponding diluition of DIG-labeled control DNA (Co-oligo), used as a reference. The membrane was analyzed by direct detection procedure (B) followed by analysis of the relative intensities to calculate the amount of DIG labeled oligonucleotide in the sample probe preparation. Data are represented as mean +/- SD.
  j. Place the membrane, oligonucleotides side facing upward, in a 254 nm UV cross-linker at a setting of 120 mJ/cm^2^ to fix the nucleic acid to the membrane. Flip the membrane (DNA side facing down) and repeat. k. Prepare 100 mL of blocking solution and allow to cool down to 20°C–23°C (∼15 min) before use.
  k. Prepare 100 mL of blocking solution and allow to cool down to 20°C–23°C (∼15 min) before use.
  l. Wash the membrane for 2 min with 2× SSC.
  m. Follow the steps described below, section: 13. d. Chemiluminescence detection, to develop the membrane and determine the probe efficiency.
  n. Compare the intensity of the probe spots to the intensity of control Oligonucleotide DIG-ddUTP labeled spots to determine the reach of full sensitivity (Figure 4B). Use the relative intensities to calculate the amount of DIG labeled oligonucleotide in the sample probe preparation needed for the hybridization step (12.d).
  o. Store labeled probes in 20 μL working aliquots at −20°C.

11. Labeling of telomere and Alu probe and determination of labeling efficiency.
  a. Add 100 pmol oligonucleotides in a DNase-RNase free PCR tube.
    ***Note:*** we suggest preparing 4 PCR tubes for each oligonucleotide and process in parallel so probe can be stored for later use.
  b. In each PCR tube, add DNase-RNase free water to make a final volume of 10 μL.
  c. Place the PCR tubes on ice and sequentially add:
    4 μL of Reaction Buffer 5× (Vial 1).
    4 μL of CoCl_2_ solution (Vial 2).
    1 μL of DIG-ddUTP solution (Vial 3).
    1 μL of Terminal transferase (400 U,Vial 4).
  d. Mix with pipetting up and down and spin down.
  e. Incubate at 37°C for 15 min in a thermocycler, then keep on ice.
  f. Add 2 μL of 0.2 M EDTA (pH 8) to stop the reaction.
  g. Add 18 μL of DNase-RNase free water to dilute the labeled oligonucleotides to a concentration of 2.5 pmol/μL.
  h. Prepare serial dilution of labeled probes and labeled control oligonucleotides from the kit (Oligonucleotide, DIG-ddUTP labeled; Vial 6) from 1:1 to 1:1000.
  i. Spot 1 μL of each serial dilution/probe along serial dilution of control oligonucleotide DIG-ddUTP labeled on a Hybond N+ positively charged nylon membrane (Figure 4A).

12. Hybridization of C-circle slot-blot membrane.
  a. Prepare 50 mL of prehybridization buffer and place it in a hybridization oven at 65°C to equilibrate.
  b. Place the cross-linked membrane onto a nylon mesh and carefully place it in a hybridization tube with the DNA side inward.
  c. Add 25 mL of prehybridization buffer and place in a rotating hybridization oven for 2 h at 65°C.
    ***Note:*** The optimal hybridization temperature is related to the GC content of the probe, homology of the probe to the target and hybridization buffer composition. If the alternative DIG Easy Hyb (Roche) or Standard Buffer containing 50% formamide are used, consider calculating an optimal hybridization temperature.
  d. Prepare hybridization solution by combining 25 mL of fresh pre-hybridization buffer with 20 μL of telomere DIG-3’end labeled probe (from a stock where probe efficiency was calculated as 2.5 pmol/μL).
  e. Equilibrate the hybridization solution in the hybridization oven at 65°C.
    ***Note:*** Concentration of 3’end labeled oligonucleotide probe in hybridization buffer is recommended between 1 – 10 pmol/mL, with a hybridization time between 6 h up to 18 h.
  f. Discard prehybridization solution and add 25 mL of pre-equilibrated hybridization solution, place the tube with the membrane in a rotating hybridization oven, incubate ∼ 18–20 h at 65°C.

13. Chemiluminescence detection
  a. Before you start, prepare 100 mL of blocking solution and allow to cool down to 20°C–23°C (∼15 min) before use.
  b. Transfer the membrane to a membrane-staining box and wash three times with 200 mL of Wash buffer 1 on a rotational shaker for 15 min.
  c. Carefully remove Wash buffer 1 and wash the membrane with 200 mL of 2× SSC for 20 min.
  d. Place the membrane in a clean heat-sealable bag and add 60 mL of blocking buffer (20°C–23°C). Seal with heat sealer avoiding the formation of air bubbles, and shake on a rotational shaker for 30 min at 20°C–23°C.
    ***Note:*** To reduce antibody amount we used a heat-sealable bag, as an alternative it is also possible to use a blot-box.
  e. Discard blocking buffer and incubate the membrane in a new heat-sealable bag with the remaining 40 mL of blocking buffer with 4 μL of anti-DIG-AP-antibody (1:10.000). Seal with heat sealer avoiding the formation of air bubbles, and shake on a rotational shaker for 1–2 h at 20°C–23°C.
  f. Transfer the membrane to a plastic tray and wash twice with 200 mL of Wash buffer 2 for 17 min on a shaker.
  g. Carefully remove Wash buffer 2, and equilibrate the membrane in 50 mL of AP buffer for 5 min.
  h. Combine 4 mL of AP buffer with 40 μl CDP-Star, protect the solution from light.
  i. Place the membrane in a clean heat-sealable bag and add the chemiluminescence substrate solution. Seal with heat sealer avoiding the formation of air bubbles.
  j. Roll a 10 mL serological pipette over the sealed bag for 5 min in the dark (DNA side facing up) and incubate for other 30 min shaking gently.
  k. Place the wet membrane, DNA side facing up, on a transparent sheet and quickly proceed with chemiluminescence detection using a ChemiDoc Imaging System or similar chemiluminescence imaging system. Troubleshooting 2, 3, 4, 5.

***Note:*** Do not use a plastic wrap to cover the blot during ChemiDoc Imaging detection. Acetate sheet or transparent film is preferable. During detection do not leave an excess of CDP-Star, as this can increase the background.

***Note:*** If the signal and the quality of images is low, the membrane can be re-incubated with CDP-Star solution for 1–2 h. The signal is stable for up to 24 h. Pay attention to completely cover the membrane with the CDP-Star substrate solution, a thin layer is enough.

***Note:*** Membranes should never be allowed to dry before stripping.

**Pause point:** The membrane can be stored in AP buffer, at +4°C for 12–24 h before proceeding with membrane stripping and re-probing.

14. Membrane stripping and re-probing.
  a. Rinse membrane thoroughly twice in autoclaved ddH_2_O for 1 min.
  b. Incubate the membrane in 50 mM NaOH stripping buffer for 30 min at 45°C.
  c. Discard stripping buffer and follow to neutralization by incubating in stripping neutralization buffer for 15 min.
  d. Rinse the membrane in 2× SSC.
    ***Note:*** Alternative stronger membrane stripping methods are: incubation in hot 0.1% SDS for 15 min with gentle agitation; or incubation with 0.2 M NaOH/0.1% SDS, 2 times 15 min at 37°C, followed by a rinse in 2**×** SSC, 5 min. We recommend not to use a higher concentration of NaOH.
  e. The completeness of stripping has to be assessed by standard exposure time in ChemiDoc Imaging or similar chemiluminescence imaging system.
    **Pause point:** The membrane can be stored in a sealed plastic bag with 2**×** SSC at 4°C.
  f. Re-probe the membrane with Alu probe following the steps described above, start from the section: 12. Hybridization of C-circle slot-blot membrane.

## Expected outcomes

ALT activity is associated with the production of C-circles, partially single-stranded extrachromosomal telomeric DNA, which serve as an ALT specific biomarker (Henson et al., 2009; Zhang et al., 2019). Amplification of C-circles present in tumor DNA using a CCA represents an improvement in ALT detection and tumor diagnosis (Grandin et al., 2019), because it is a robust and specific assay, and requires a low amount of genomic DNA (Henson et al., 2009, 2017; Cesare and Griffith, 2004). In the protocol described here, we report a detailed method for quantitative measurement of C-circles from FFPE specimen material providing appropriate implementation and controls. Given the value of biopsy specimens, our protocol describes a genomic DNA isolation procedure from 3–8 FFPE sections with a thickness of 5 μm and typically gives rise to 70–300 ng of genomic DNA, depending on the nature of the sample.

**Figure 5:**
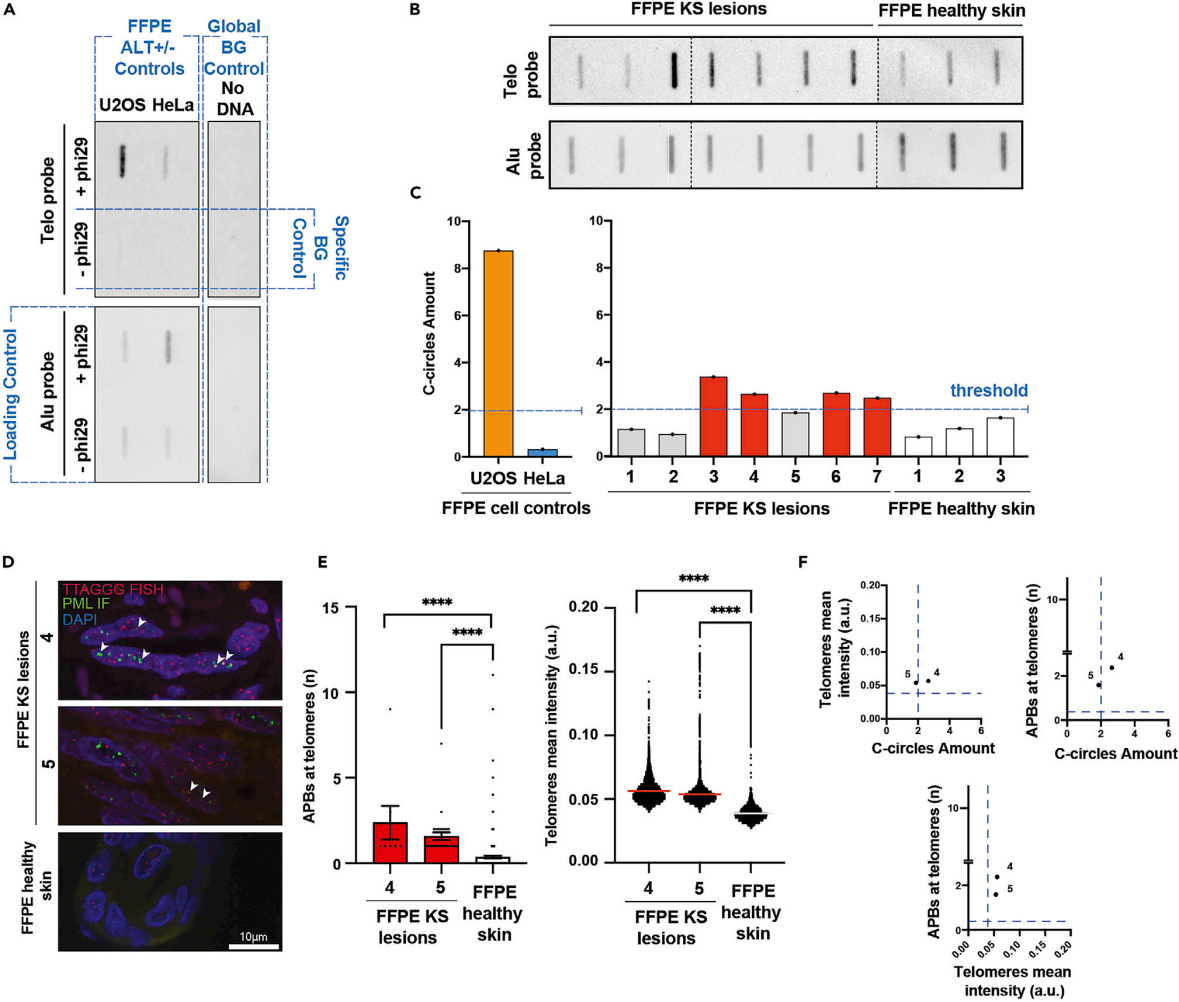
Expected outcomes and analysis (A) Representative slot-blot detected from the telomere and Alu probed-membrane (loading control), showing FFPE ALT positive (U2OS) and ALT negative (HeLa) controls with specific background (BG) (-Phi29) and global background (BG) control (No DNA). (B) Representative slot-blot detected from the telomere and Alu probed-membrane (loading control) showing FFPE KS lesions samples and FFPE healthy skin samples. (C) Histograms of the CCA values analyzed from the slot-blots in (A) and (B). (D–F) An example of additional ALT assays can be performed to confirm ALT activity and exclude possible false-negative samples. (D and E) (D) Representative fluorescent microscope images and (E) quantification of ALT-associated PML bodies (APBs) and mean telomere signal intensity of the samples 4- and 5-FFPE KS lesions (with C-circles amount close to the 2-fold threshold) and control FFPE healthy skin. Antibody against PML bodies was used combined with telomere-FISH and counterstained with DAPI. Statistical significance was tested with a non-parametric Mann-Whitney U test (∗∗∗∗p < 0.0001). Data are represented as mean +/− SEM. Scale bar: 10 μm. (F) Scatterplots showing the relationship between ALT assays for samples 4 and 5 (FFPE KS lesions). Dotted line reflects the threshold as determined by analysis of FFPE healthy skin.

A successful and robust assay should show a clear increase of C-circle levels in ALT positive tumors that can be correlated with ALT+ control samples (Figures 5A–5C). This is dependent on the C-circle levels relative to genomic DNA and telomere length, amplification of C-Circles with an RCA reaction and sequence-specific detection of the amplification products by native telomeric slot-blot.

## Quantification and statistical analysis

According to the instrument used for chemiluminescence detection, quantitation and analysis of the slot-blot should be performed with an image processing software such as Image Lab™ Software (BioRad) (https://www.bio-rad.com/webroot/web/pdf/lsr/literature/10000076953.pdf) or ImageJ (https://imagej.net/Fiji/Downloads), where the original file format can be used to quantify the mean value intensity of each dot.

We suggest to evaluate the mean value intensity of each dot in the membrane incubated with telomere and repetitive Alu elements probe, for the following samples:

FFPE tumor samples with specific background controls (with and without Phi29 polymerase)

FFPE normal tissue samples with specific background controls (with and without Phi29 polymerase)

FFPE ALT positive (U2OS) and ALT negative (HeLa) controls with specific background controls (with and without Phi29 polymerase)

Global background control (DNA omitted).

1. Determination of CCA signal (Figures 5A–5C)
  a. Start with the intensity quantification of each dot detected from the telomere probed-membrane.
  b. Subtract the value from the global background control (DNA omitted) to each value from the samples (with and without Phi29 polymerase).
  c. Subtract the value from specific background controls (without Phi29 polymerase) to each relative value from the samples (with Phi29 polymerase).
  d. Correct the resulting values to the loading control, by dividing the telomere intensity values from (c) by their respective Alu repeats probe signal (of samples with Phi29 polymerase and previously substracted the value of the global background control).
  e. If the comparison between multiple slot-blot membranes is needed, use the amount of C-circles levels relative to positive (U2OS) controls in the different blots, to normalize the signal of each specimen.
2. Considerations of ALT status (Figure 5C):
  a. Plot in a histogram the CCA values for: FFPE ALT positive (U2OS) and ALT negative (HeLa) controls, FFPE tumor and normal tissue samples.
  b. To verify the success of the assay, consider the CCA level in FFPE HeLa negative and U2OS positive control, generally their CCA levels are between indistinguishable / less than two-fold of the background and higher than five-fold of the background, respectively.
  c. Consider tissue specimens as an ALT negative sample when the CCA levels are less than two-fold of the background, according to the FFPE HeLa negative control signal.
  d. Consider the CCA values in FFPE normal tissue samples and evaluate the possible small amount of ALT activity relative to the tissue. Set up a threshold no lower than two-fold of the background signal.
  e. ALT activity was considered significant if the CCA level is generally up to two-fold of the background (when all the controls were considered). The threshold may be set up higher than two-fold of the background signal, when the FFPE normal tissue display CCA higher values due to small amount of ALT activity relative to the tissue.

***Note:*** Our experience is that FFPE tumour specimens will have lower values than FFPE U2OS positive control signal.

***Note:*** To confirm ALT activity and the exclusion of possible false-negative samples as a consequence of formalin associated DNA fragmentation, we recommend performing additional ALT assays (Figures 5D–5F).

## Limitations

FFPE tissue from biopsy specimens represents the largest available source of biological material for experimental research and diagnostic study. In the last few years, their value in molecular oncologic testing is increasing, due to new technologies used to obtain high-quality RNA and whole-genome sequencing (WGS) analysis that can be used to predict patient responses and outcomes. Here, we present a protocol to determine the presence of C-Circles, a specific hallmark of ALT, in FFPE sections, that can be used to diagnose and understand ALT mechanisms in tumors. FFPE tissues may be stored for a long time and nucleic acids may be recovered from them for a significant time after fixation (Groelz et al., 2018). Nevertheless, one limitation of the use of FFPE samples for this protocol is related to the process of fixation, where prolonged formalin fixation can lead to cross-linking, degradation and DNA fragmentation (Gilbert et al., 2007). These alterations inevitably affect the quality and quantity of DNA extracted from FFPE tissue, can generate increased false negative rates and result in low levels of CCA signal. For this reason, particularly if the nature of the starting material is unknown, we recommend performing all the controls described in the protocol and correlate the ALT status of the samples to additional ALT assays (Gunkel et al., 2017; Lippert et al., 2021; Henson et al., 2005). Another limitation to consider is related to the intra-tumoral heterogeneity of the biopsies material, where the ALT+ cancer cells may occur within the context of a telomerase positive tumor and normal soma. Tumor cellularity before sample processing should be assessed to ensure the presence of >70% tumor cells.

## Troubleshooting

### Problem 1

Low DNA concentration after FFPE Genomic DNA isolation (visible in: Quantification of genomic DNA, step: 8. i)

### Potential solution

Low DNA concentration can be caused by inefficient cell lysis. Residual formalin can inhibit the proteinase K digestion; excess of paraffin must be removed before DNA isolation using a scalpel (step: Before you begin - Preparation of sections from FFPE tissue specimen blocks - 10).

Repeat the procedure using fresh proteinase K (step: Isolation of genomic DNA from FFPE sections - 2. a) and/or using fresh cut sections, discard sections that were exposed to air (step: Before you begin - Preparation of sections from FFPE tissue specimen blocks - 10).

Check the temperature of the Formalin crosslink reversal (at 90°C), this step allows to partially remove formalin crosslinking of the released DNA and improve the DNA yields. Make sure not to exceed the recommended temperature and the incubation time (step: Isolation of genomic DNA from FFPE sections - 3. a).

The degree of fragmentation depends on the type and age of the sample and the conditions used for fixation. Use a fixation time of 16–24 h (step: Before you begin - Cell processing and slide preparation FFPE cell-pellets – 5. e).

Make sure the ethanol used to remove residual xylene from the samples and to perform DNA isolation was 96%–100%. When the bottle is opened very frequently, the ethanol can present low-percentage. Change the bottle when it happens (step: Isolation of genomic DNA from FFPE sections – 1. f).

Make sure to remove any residual ethanol before DNA elution (step: Isolation of genomic DNA from FFPE sections – 5. m).

### Problem 2

No or low signal observed after slot-blot detected with DIG-labeled telomere probes (visible in: Non-radioactive detection of C-circle blotted products, step 13. k).

### Potential solution

One possible reason could be related to decreased phi29 DNA polymerase amplification efficiency. Make sure that phi29 polymerase has been properly stored at −20°C for no longer than 24 months.

Make sure the enzyme and DTT used for the reaction buffer have been properly stored at −20°C. Discard old reaction buffer stocks or DTT stocks that have been repeatedly frozen and thawed (step: Before you begin, 2 - 3 / step: Rolling Circle Amplification 9. a-c / Materials and Equipment - Rolling Circle Master Mix (RCMM)).

RCA reactions require the circular telomeric C-strand template to be complete. Factors that may cause DNA nicks and/or DNA degradation should be avoided. Use always sterile disposable material, DNase-RNase free microtubes and ddH_2_O, autoclave all the solutions, store DNA in a buffer such as 10 mM Tris pH 7.6 to avoid acid hydrolysis, store the DNA at −80°C or a freezer without a defrost cycle, avoid multiple freeze-thaw cycles and perform all the preparation of RCA reaction on ice.

It is possible that the assay was performed with an insufficient amount of DNA template in the sample. Check again the DNA concentration of the sample (step: Quantification of genomic DNA). If the DNA concentration is confirmed, increase the ng of DNA in the RCA reaction (step: Rolling Circle Amplification – 9. b). Perform a control test, using a serial dilution of genomic DNA from the samples and the ALT+ cell line to check the linearity of the C-circles levels in the function of ng of genomic DNA.

Make sure the probe used for the hybridization step was correctly checked for labeling efficiency (step: Non-radioactive detection of C-circle blotted products – Labeling of telomere and Alu probe and determination of labeling efficiency – 11. h-n / Figure 4).

Increase the incubation time with CDP-Star solution for 1–2 h, the signal is stable up to 24 h (step: Non-radioactive detection of C-circle blotted products – 13. j).

Increase the exposure time during chemiluminescence slot-blot detection (step: Non-radioactive detection of C-circle blotted products – 13. k).

### Problem 3

No signal observed for some samples after slot-blot detection with DIG-labeled Alu probes (visible in: Non-radioactive detection of C-circle blotted products, step 13. k).

### Potential solution

Ensure there are no air bubbles when you load the samples for a native slot-blot. Use a roller to remove bubbles when you assemble the slot-blot with two Whatman papers and membrane on top. Do not allow the membrane to dry (step: Transfer of RCA products to membrane – 10. h).

### Problem 4

High membrane background after slot-blot detection (visible in: Non-radioactive detection of C-circle blotted products, step 13. k).

### Potential solution

This problem can be caused by excessive telomere probe concentration or insufficient membrane washes after hybridization and/or antibody incubation passage. Try to decrease the telomere probe concentration in the hybridization solution and increase by 2 min each of the wash steps after hybridization and antibody detection steps. Make sure to use an appropriate volume of wash buffer related to membrane size (step: Non-radioactive detection of C-circle blotted products – 12. / 13.).

### Problem 5

Not-homogeneous signal and/or membrane background after slot-blot detection (visible in: Non-radioactive detection of C-circle blotted products, step 13. k).

### Potential solution

Check the functionality of the rotating mode of the hybridization oven (step: Non-radioactive detection of C-circle blotted products – 12. c - f).

## Resource availability

### Lead contact

Simon Boulton: simon.boulton@crick.ac.uk

### Materials availability

This study did not generate new unique reagents.

### Data and code availability

This study did not generate datasets.
